# Supplementary material for: Transcription factor TFCP2L1 patterns cells in the mouse kidney collecting ducts
Source: eLife. 2017 Jun 3;6:e24265. doi: 10.7554/eLife.24265 (PMC5484618; doi:10.7554/eLife.24265)
Supplement: Supplementary file 5. — DOI: http://dx.doi.org/10.7554/eLife.24265.029 [file elife-24265-supp5.docx]

| Target | Company (Catalogue Number) RRID | Dilution |
| --- | --- | --- |
| Tfcp2l1 | R&D (AF5726) RRID:AB_2202564 | 200 |
| Krt8 | DSHB (TROMAI) RRID:AB_531826 | 200 |
| Aqp2 | Sigma-Aldrich (A7310) RRID:AB_476762 | 500 |
| Atp6v1b1/b2 | Santa-Cruz Biotech (SC-20943) RRID:AB_677577 | 200 |
| Cdh1 | BD Biosciences (610181)RRID:AB_397580 | 500 |
| Calb1 | Sigma-Aldrich (C9848) RRID:AB_2314067 | 200 |
| Jag1 | R&D (1277) RRID:AB_354713, DSHB (TS1.15H) RRID:AB_528317 | 200 |
| Nkcc2 | Sigma-Aldrich (AV41388) RRID:AB_1854505 | 200 |
| Gfp | Novus Biological (NB100-1614) RRID:AB_10001164 | 500 |
| Pendrin | Santa-Cruz Biotech (sc-50346) RRID:AB_2190490 | 200 |
| Ae-1 | Alpha-Diagnostics (AE11-A) RRID:AB_1609266 | 200 |
| Aqp4 | Alomone Labs(AQP-004) RRID:AB_2039734 | 200 |
| Aqp3 | Alomone Labs (AQP-003) RRID:AB_2039731 | 200 |
| Notch1 | R&D (AF1057) RRID:AB_2153372 | 200 |
| Foxi1 | Abcam (ab20454) RRID:AB_732416 | 200 |
| Activated Notch1 | Abcam (ab8925) RRID:AB_306863 | 200 |
| Numb | Abcam (ab14140) RRID:AB_443023 | 200 |
| Hes1 | Santa-Cruz Biotech (sc-25392) RRID:AB_647996 | 200 |
| Podocalyxin-like 1 | Santa-Cruz Biotech (sc-33140) RRID:AB_2166110 | 200 |
| Romk1 | Alomone Labs (APC-001) RRID:AB_2040105 | 200 |
| Ca2 | Abcam (ab8953) RRID:AB_306884 | 100 |

Antibodies

Primers

| Name | Sequence (5’-3’) | Usage |
| --- | --- | --- |
| mTfcp2l1-F1 | GGTTGCCTGTATGTCTGTAC | Genotyping mTfcp2l1 floxed locus, forward primer |
| mTfcp2l1-L2 | CAAGGTAAGGTGTCTCACTG | Genotyping mTfcp2l1 floxed locus, reverse primer |
| mJag1-F | TCAGGCATGATAAACCCTAGC | Genotyping mJag1 floxed locus, forward primer |
| mJag1-R | CTACATACAGCATCTACATGC | Genotyping mJag1 floxed locus, reverse primer |
| Cre-F | GGACATGTTCAGGGATGGCCAGGCG | Genotyping, Cre, forward primer |
| Cre-R | GCATAACCAGTGAAACAGCATTGCTG | Genotyping, Cre, reverse primer |
| TomatoWT-F | CTCTGCTGCCTCCTGGCTTCT | Genotyping, Rosa26Tomato, WT forward primer |
| TomatoWT-R | CGAGGCGGATCACAAGCAATA | Genotyping, Rosa26Tomato, WT reverse primer |
| TomatoTG-R | TCAATGGGCGGGGGTCGTT | Genotyping, Rosa26Tomato, TG reverse primer |
